# Television viewing, computer use, obesity, and adiposity in US preschool children

**DOI:** 10.1186/1479-5868-4-44

**Published:** 2007-09-25

**Authors:** Jason A Mendoza, Fred J Zimmerman, Dimitri A Christakis

**Affiliations:** 1 USDA/ARS Children's Nutrition Research Center and Academic General Pediatrics, Department of Pediatrics; and the Dan L. Duncan Cancer Center; Baylor College of Medicine, Houston, TX, USA; 2 Child Health Institute, University of Washington, Seattle, WA, USA; 3 Department of Health Services, University of Washington, Seattle, WA, USA; 4 Department of Pediatrics, University of Washington and Children's Hospital and Regional Medical Center, Seattle, WA, USA

## Abstract

**Background:**

There is limited evidence in preschool children linking media use, such as television/video viewing and computer use, to obesity and adiposity. We tested three hypotheses in preschool children: 1) that watching > 2 hours of TV/videos daily is associated with obesity and adiposity, 2) that computer use is associated with obesity and adiposity, and 3) that > 2 hours of media use daily is associated with obesity and adiposity.

**Methods:**

We conducted a cross-sectional study using nationally representative data on children, aged 2–5 years from the National Health and Nutrition Examination Survey, 1999–2002. Our main outcome measures were 1) weight status: normal versus overweight or at risk for overweight, and 2) adiposity: the sum of subscapular and triceps skinfolds (mm). Our main exposures were TV/video viewing (≤ 2 or > 2 hours/day), computer use (users versus non-users), and media use (≤ 2 or > 2 hours/day). We used multivariate Poisson and linear regression analyses, adjusting for demographic covariates, to test the independent association between TV/video viewing, computer use, or overall media use and a child's weight status or adiposity.

**Results:**

Watching > 2 hours/day of TV/videos was associated with being overweight or at risk for overweight (Prevalence ratio = 1.34, 95% CI [1.07, 1.66]; n =1340) and with higher skinfold thicknesses (β = 1.08, 95% CI [0.19, 1.96]; n = 1337). Computer use > 0 hours/day was associated with higher skinfold thicknesses (β = 0.56, 95% CI [0.04, 1.07]; n = 1339). Media use had borderline significance with higher skinfold thicknesses (β = 0.85, 95% CI [-0.04, 1.75], P=0.06; n = 1334)

**Conclusion:**

Watching > 2 hours/day of TV/videos in US preschool-age children was associated with a higher risk of being overweight or at risk for overweight and higher adiposity–findings in support of national guidelines to limit preschool children's media use. Computer use was also related to higher adiposity in preschool children, but not weight status. Intervention studies to limit preschool children's media use are warranted.

## Background

The epidemic of childhood obesity is a major public health problem in the US, where in 2003–2004, 26.2% of children aged 2–5 years, 37.2% of children aged 6–11 years, and 34.3% of adolescents 12–19 years were at risk for overweight or overweight [1]. Some large epidemiological studies and one recent meta-analysis have found positive associations between television viewing and childhood obesity [2-4]. Previous intervention studies in school-age children have supported television and video viewing as causes of childhood obesity [5,6]. In response to the growing problem of childhood obesity and other health issues associated with television viewing, the American Academy of Pediatrics (AAP) has issued national guidelines for parents to limit their children's total media time (with entertainment media) to no more than 1 to 2 hours of quality programming per day for children 2 years of age and older [7-9].

Television viewing is the most popular form of media use among young children [10]. Some studies have linked television viewing to excess weight gain in preschool children [11-13]. However, these studies had limitations. First, they measured television/video viewing but not other forms of media such as computer use. Moreover, some did not specifically test the AAP's 2-hour/day cut-off with regard to media time and weight status [11,12], had samples limited to a specific age or geographic areas [11,12], reported race/ethnicity as white or not white [13], or related television/video viewing to BMI but not other forms of adiposity [11-13]. While BMI is the recommended method for population-based screening of children for obesity, it was a poor predictor of body fat for individual children [14]. Other measures, such as skinfold thicknesses, were highly correlated with adiposity, [15] lipids [16], and insulin [16] in children, and thus may provide additional useful information [17].

The AAP recommendation is not specific to television, but instead was written in terms of overall media use or what some call "screen time." At the time the initial recommendations were established, computer use among preschoolers was very limited. That has now changed. Computer usage is rapidly gaining in popularity among toddlers and preschool children. A series of Kaiser Family Foundation studies reported that 4–27% of children less than 6 years of age used a computer on the assessment day for an average of almost 1 hour [10,18,19]. Like television viewing, computer use may lead to decreased time spent being physically active, which may predispose to excess weight gain. However to our knowledge, the relationship between computer use and weight status in US preschool children has not been previously described.

The AAP's recommendation to limit media time is a national one, which underscores the importance for testing it on a nationally representative sample of preschool children, aged 2–5 years. Moreover, because television viewing and obesity differ by race/ethnicity and socioeconomic status [12,13], it is also important to examine this relationship using nationally representative data to ensure adequate numbers of minority and low-income subjects of differing urbanization types and regions of the country.

The main objective of this study was to test three hypotheses using nationally representative data on subjects aged 2–5 years from the National Health and Nutrition Examination Survey (NHANES) 1999–2002: 1) whether watching greater than two hours of television daily is independently associated with obesity (overweight or at risk for overweight) or adiposity (the sum of subscapular and triceps skinfolds), 2) whether computer use is independently associated with obesity or adiposity, and 3) whether overall media use (television/video viewing plus computer use) greater than two hours daily is independently associated with obesity or adiposity, on a population level. We analyzed television viewing and computer use together because the AAP recommendation refers to media time and therefore encompasses both of these types of media use. We also analyzed them separately because the relationship between television viewing and obesity is well studied, while the relationship between computer use and obesity is not. For example, television use and its relationship to obesity is likely mediated by a number of factors such as 1) displacement of physical activity, 2) advertisements which encourage selection and consumption of low-nutrient, high caloric foods, and 3) increased dietary intake or snacking. In contrast, it is currently unknown whether computer use in preschoolers is associated with weight status.

## Methods

### Data source

The NHANES is a series of cross-sectional surveys conducted by the Centers for Disease Control and Prevention (CDC), which serves as one of the key measures for Healthy People 2010 [20]. We used NHANES 1999–2002, the latest, fully released version of NHANES, to obtain a nationally representative sample of the US non-institutionalized civilian population through its complex, stratified, multistage, probability cluster sampling design. Most subjects were interviewed in-person although a small sub-sample was interviewed over the telephone. For children less than 6 years of age, proxy interviews were conducted. NHANES methods have been reported in detail elsewhere [21]. This study was reviewed and deemed exempt by the University of Washington Human Subjects Division.

### Subjects

For this analysis, we chose all children, aged 2 to 5 years (n = 1809). Subjects with missing data were excluded from analyses and the corresponding sample size is given for each analysis.

### Outcome variables

Height, weight, triceps skinfold thickness, and subscapular skinfold thickness were obtained using standardized techniques and equipment [22]. Body mass index (BMI) was calculated as weight (kilograms) divided by the square of height (meters^2^) and their corresponding BMI percentiles were calculated from the CDC growth charts [23]. Triceps and subscapular skinfolds were summed into one measure to provide a more global index of adiposity. Children were also classified as underweight (< 5^th ^%) according to World Health Organization guidelines [24], or normal weight (≥ 5^th ^and < 85^th ^%), at risk for overweight (≥ 85^th ^and < 95^th ^%), and overweight (> 95^th ^%), according to guidelines from the Centers for Disease Control and Prevention [23]. For the purposes of the multivariate Poisson regression models, we dichotomized children into two categories by weight status: 1) normal weight (≥ 5^th ^and < 85^th ^% for age and gender) and 2) at risk for overweight or overweight (> 85^th ^% for age and gender). Underweight children were excluded from the multivariate analyses (n = 66).

### Main exposure

Television/video viewing was a categorical variable and was assessed similarly to previous reports from older releases of NHANES [2,25], by the following in which "SP" refers to sample person: "About how many hours did (SP) sit and watch TV or videos yesterday? Would you say less than 1 hour, 1 hour, 2 hours, 3 hours, 4 hours, 5 hours or more, or none?" In order to test the AAP guidelines in the multivariate analyses, we dichotomized TV/video viewing into two categories: 2 hours or less/day and greater than 2 hours/day. Computer use was also a categorical variable and similarly assessed using the following: "About how many hours did (SP) use a computer or play computer games yesterday? Would you say less than 1 hour, 1 hour, 2 hours, 3 hours, 4 hours, 5 hours or more, or none?" Because computer use was expected to be low among preschoolers, we also dichotomized the variable to 0 hours/day and greater than 0 hours/day to classify children as non-users and users, respectively. Because we were interested in assessing preschoolers' media time, we combined the television/video viewing and computer use variables into one measure, henceforth termed media use. In combining the categorical variables, we took a conservative approach and classified those participants who reported less than 1 hour of television or computer use as having none.

### Covariates

We adjusted for several covariates that might confound the relationship between TV/video viewing or computer use and our outcomes of interest. Socioeconomic and demographic variables were reported as follows: 1) Gender, 2) Age as a continuous variable, 3) Race/ethnicity categorized as non-Hispanic white, non-Hispanic black, Mexican-American, and Other; and 4) Household income reported as the poverty to income ratio (PIR), which is the ratio of income to the family's appropriate poverty threshold as determined by the US Census Bureau [26]. PIR values less than 1 are below the poverty threshold, which is adjusted annually for inflation with the Consumer Price Index. PIR was provided by NHANES in the following six categories: < 1, ≥ 1 < 2, ≥ 2 < 3, ≥ 3 < 4, ≥ 4 < 5, and ≥ 5 PIR [21].

### Statistical analyses

We used the Pearson chi-squared statistic to test for 1) differences in the proportions of demographic variables and main exposures of television/video viewing, computer use, or overall media use by weight status; 2) differences in the proportions of television/video viewing, computer use, or overall media use by socio-demographic covariates; and 3) differences in proportions of television/video viewing versus computer use. We used a series of multivariate Poisson regression models to determine the independent association between TV/video viewing (n = 1340), computer use (n = 1340), or media use (n = 1337), and a child's weight status, adjusting for gender, age, race/ethnicity, and household income. We also used a similar series of multivariate linear regression models, controlling for socio-demographic variables, to determine the independent association between TV/video viewing (n = 1337), computer use (n = 1339), or media use (n = 1334), and the measure of adiposity: the sum of subscapular and triceps skinfold thicknesses. Subjects with missing data were dropped from each of the bivariate and multivariate regression models. Demographic differences between dropped subjects and those included in the multivariate Poisson regression model were tested by the Pearson chi-squared statistic.

Stata version 9 was used for all analyses (StataCorp LP, College Station, TX). Survey estimation commands for complex survey data were used in the analyses taking into account weighted observations and the probability of selection, nonresponse, and post-stratification adjustments, to obtain representative estimates of US children 2 to 5 years old. A significance level of 0.05 was used for all analyses. We present means and standard errors (means +/- standard errors) unless otherwise indicated. Taylor series linearization was used to estimate standard errors.

## Results

Average age was 3.5 years +/- 0.03 years (SE) and 51.8% +/- 2.1% were female. Sample sizes with their corresponding estimates of percentages for gender, race/ethnicity, income, media use, television viewing, and computer use by overweight status are given in Table 1. An estimated (uncorrected) 22.0% +/- 1.5% were overweight or at risk for overweight. Both media use and television/video viewing were associated with weight status in the bivariate analyses.

**Table 1:** Demographics and key exposures of participants, aged 2–5 years, from NHANES 1999–2002 by weight status.*

	Normal weight	At risk for overweight or overweight	Total
Gender			
Female	51.8 +/- 2.4	51.8 +/- 3.5	51.8 +/- 2.1
Male	48.3 +/- 2.4	48.2 +/- 3.5	48.2 +/- 2.1
Race/Ethnicity			
Non-Hispanic white	63.6 +/-2.8	56.1 +/- 4.2	62.0 +/- 2.7
Non-Hispanic black	13.9 +/- 2.1	15.1 +/- 2.8	14.2 +/- 2.1
Mexican-American	12.6 +/- 1.8	14.9 +/- 2.0	13.1 +/- 1.6
Other	9.9 +/- 1.8	13.9 +/- 2.9	10.8 +/- 1.8
Poverty Income Ratio			
< 1	26.4 +/- 1.7	25.0 +/- 2.7	26.1 +/- 1.5
≥ 1 < 2	24.1 +/- 2.1	33.3 +/- 5.5	26.1 +/- 2.4
≥ 2 < 3	15.5 +/- 1.7	15.3 +/- 3.2	15.5 +/- 1.7
≥ 3 < 4	13.3 +/- 1.7	6.8 +/- 2.0	11.9 +/- 1.4
≥ 4 < 5	8.3 +/- 1.5	5.5 +/- 2.1	7.7 +/- 1.4
≥ 5	12.4 +/- 1.8	14.1 +/- 3.7	12.7 +/- 1.8
Television/video use^‡^			
≤ 2 hours/day	70.7 +/- 2.0	60.8 +/- 3.8	68.6 +/- 2.1
> 2 hours/day	29.3 +/- 2.0	39.2 +/- 3.8	31.4 +/- 2.1
Computer use			
0 hours/day	45.6 +/- 2.2	39.9 +/- 3.7	44.3 +/- 2.1
< 1 hour/day	37.9 +/- 2.0	39.2 +/- 3.8	38.2 +/- 1.9
1 hour/day	11.2 +/- 0.9	12.0 +/- 2.3	11.4 +/- 0.8
2 hours/day	3.3 +/- 0.7	6.4 +/- 1.9	4.0 +/- 0.7
3 hours/day	1.1 +/- 0.4	0.9 +/- 0.4	1.0 +/- 0.3
4 hours/day	0.3 +/- 0.2	0.7 +/- 0.7	0.4 +/- 0.2
≥ 5 hours/day	0.6 +/- 0.3	0.9 +/- 0.7	0.7 +/- 0.3
Media use^†^			
≤ 2 hours/day	64.4 +/- 2.0	56.7 +/-3.8	62.7 +/- 2.0
> 2 hours/day	35.6 +/- 2.0	43.3 +/-3.8	37.3 +/- 2.0

* Estimated percentages +/- standard errors are given. n = 1340 for gender, race/ethnicity, poverty income ratio, and TV/video use; n = 1337 for computer use and media use. Overall, 22.0% +/- 1.5% were overweight or at risk for overweight.

^† ^P = 0.026 for the Pearson chi-squared statistic

^‡^P = 0.0021 for the Pearson chi-squared statistic

**Figure 1:**
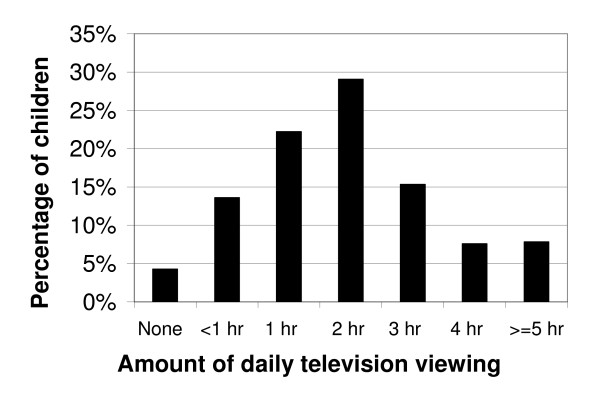
Percentage of US children, aged 2–5 years, by the amount of daily television/video viewing (n = 1796).

**Figure 2:**
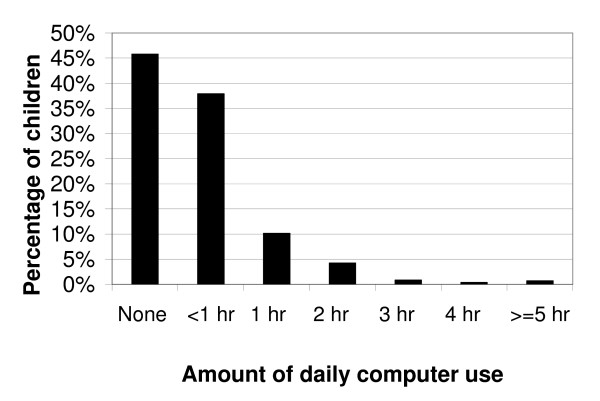
Percentage of US children, aged 2–5 years, by the amount of daily computer use (n = 1799).

**Figure 3:**
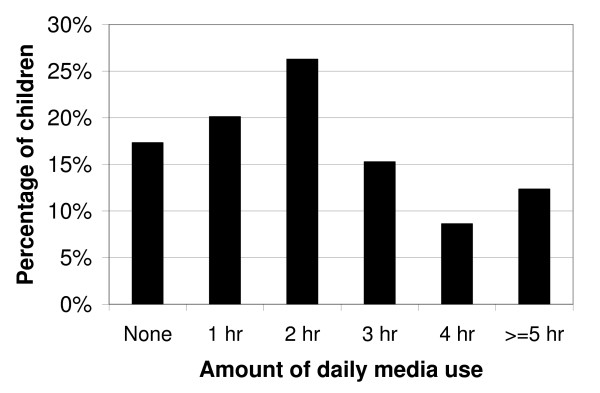
Percentage of US children, aged 2–5 years, by the amount of daily media use (n = 1792).

Television/video viewing was the more prevalent form of media use, compared to computer use (Figures 1 and 2). With regard to the AAP recommendations for limiting media use, 30.8% +/- 2.0% of US preschool children exceeded the guidelines by television viewing alone. Most children watched between 1–3 hours of TV/videos on the assessment day. Exceeding the AAP recommendations by television/video viewing alone was associated with higher age and poverty status (P < 0.05, Table 2). Non-Hispanic blacks and "Other" race preschoolers had the highest percentage who exceeded the recommendations when only considering television/video viewing (P < 0.05, Table 2). In contrast, most preschool children used the computer for less than 1 hour on the assessment day, or not at all (P < 0.05, Figure 3). For instance, while only 4.3% +/- 1.2% of children watched no TV/videos on the assessment day, 45.8% +/- 1.9% of children did not use a computer on the assessment day. Preschool children who were older or from families with higher incomes were more likely to have used a computer on the assessment day (P < 0.05, Table 3). Non-Hispanic black children were more likely to have used a computer than their white peers, while Mexican-American children were less likely to have used a computer on the assessment day (P < 0.05, Table 3). We found no significant differences by gender (P > 0.05, Table 3). In combining television/video viewing and computer use, we report that overall media use was prevalent among the 2–5 year old participants and approximately 36.2% +/- 1.9% exceeded the AAP recommendations with this combined exposure (Figure 3).

**Table 2:** Characteristics of participants, aged 2–5 years, from NHANES 1999–2002 by daily television/video viewing exposure.*

	≤ 2 hours	> 2 hours
Gender		
Male	68.2 +/- 3.0	31.8 +/- 3.0
Female	70.2 +/-2.1	29.8 +/-2.1
Age†		
2 years	77.1+/- 2.0	22.9 +/- 2.0
3 years	68.2 +/- 3.1	31.8 +/- 3.1
4 years	62.0 +/- 2.8	38.0 +/- 2.8
5 years	68.9 +/- 3.3	31.1 +/- 3.3
Race/ethnicity‡		
Non-Latino White	71.9 +/- 2.4	28.1 +/- 2.4
Non-Latino Black	58.4 +/- 3.2	41.6 +/- 3.2
Mexican-American	72.5 +/- 2.1	27.5 +/- 2.1
Other	64.6 +/- 5.3	35.4 +/- 5.3
Poverty Income Ratio§		
0 < 2	63.3 +/- 2.9	36.8 +/- 2.9
≥ 2 < 4	75.7 +/- 2.5	24.3 +/- 2.5
≥ 4	75.0 +/- 4.8	25.0 +/- 4.8

* Estimated percentages +/- standard errors are given. n = 1796, subjects with missing data were excluded.

† P = 0.0003 for the Pearson chi-squared statistic

‡ P = 0.002 for the Pearson chi-squared statistic

§P = 0.013 for the Pearson chi-squared statistic

**Table 3:** Characteristics of participants, aged 2–5 years, from NHANES 1999–2002 by computer exposure.*

	0 hours	> 0 hours
Gender		
Male	45.6 +/- 2.9	54.4 +/- 2.9
Female	46.0 +/- 2.2	54.0 +/- 2.2
Age†		
2 years	59.8 +/- 3.1	40.2 +/- 3.1
3 years	48.7 +/- 3.5	51.3 +/- 3.5
4 years	39.7 +/- 2.6	60.3 +/- 2.6
5 years	33.1 +/- 3.1	66.9 +/- 3.1
Race/ethnicity‡		
Non-Latino White	47.8 +/- 3.0	52.2 +/- 3.0
Non-Latino Black	36.8 +/- 2.1	63.2 +/- 2.1
Mexican-American	52.6 +/- 3.1	47.4 +/- 3.1
Other	38.5 +/- 6.0	61.6 +/- 6.0
Poverty Income Ratio§		
0 < 2	51.1 +/- 2.4	48.9 +/- 2.4
≥ 2 < 4	42.2 +/- 3.3	57.8 +/- 3.3
≥ 4	38.1 +/- 4.1	61.9 +/- 4.1

* Estimated percentages +/- standard errors are given. n = 1799, subjects with missing data were excluded.

† P < 0.0001 for the Pearson chi-squared statistic

‡ P = 0.044 for the Pearson chi-squared statistic

§P = 0.011 for the Pearson chi-squared statistic

In comparing television/video viewing to computer use exposures (Table 4), higher television/video viewing was significantly associated with more computer use (P < 0.0001), although computer use was generally modest for every level of television/video exposure. Most preschool children, including those that watched 4 or more hours on the assessment day, spent 1-hour or less on the computer. Computer use did not appear to displace television/video use since these exposures were positively correlated.

**Table 4:** Preschool children's television/video viewing versus computer use exposures.*

	Computer use					
Television/video viewing	< 1 hour/day	1 hour/day	2 hours/day	3 hours/day	4 hours/day	≥ 5 hours/day

< 1 hour/day	96.6 +/- 1.7	2.1 +/- 1.2	1.3 +/- 0.7	0	0	0
1 hour/day	88.8 +/- 1.8	9.7 +/- 1.8	1.2 +/- 0.7	0.3 +/- 0.2	0	0
2 hours/day	82.4 +/- 2.2	12.1 +/- 1.9	4.8 +/- 1.2	0.5 +/- 0.4	0.1 +/- 0.1	0.1 +/- 0.1
3 hours/day	74.8 +/- 3.1	11.6 +/- 2.0	9.3 +/- 2.3	3.3 +/- 1.6	1.0 +/- 0.8	0
4 hours/day	70.8 +/- 4.8	18.3 +/- 4.5	6.6 +/- 2.4	1.2 +/- 0.4	2.5 +/- 1.7	0.5 +/- 0.5
≥ 5 hours/day	73.3 +/- 3.7	12.8 +/- 2.9	5.2 +/- 2.1	0.7 +/- 0.5	0	8.0 +/- 2.6

* Estimated percentages +/- standard errors are given. n = 1792, P < 0.0001 for the Pearson chi-squared statistic.

The mean age of participants excluded from the multivariate Poisson regression models due to missing data (3.1 years, 95% CI [3.0, 3.3]) was slightly younger than the age of those with complete data (3.5 years 95% CI [3.5, 3.6]). However, participants did not differ with regard to gender, race/ethnicity, and poverty to income ratio (P > 0.05).

From the multivariate Poisson regression model, adjusting for age, gender, race/ethnicity, and income: compared to children who watched 2 hours or less of TV/videos on the assessment day, those who watched greater than 2 hours were more likely to be overweight or at risk for overweight (Table 5, Prevalence ratio = 1.34, 95% CI [1.07, 1.66], P = 0.01). Moreover, only TV viewing, and not covariates such as race/ethnicity or income, was significantly associated with weight status. From the multivariate linear regression model, watching more than 2 hours of television on the assessment day was also associated with higher skinfold thicknesses (Table 5, β = 1.08, 95% CI [0.19, 1.96], P = 0.02). Female gender was also associated with higher skinfold thicknesses (Table 4).

**Table 5:** Television viewing as a correlate for overweight status and skinfold thicknesses (mm).*

	Overweight Status^†^			Skinfolds^§^		
	Prevalence Ratio	[95%	CI]	β	[95%	CI]

Television viewing						
≤ 2 hrs/day	Reference			Reference		
> 2 hrs/day	**1.34**	**1.07**	**1.66**	**1.08**	**0.19**	**1.96**
Age (years)	1.13	0.99	1.30	0.07	-0.37	0.52
Gender						
Male	Reference			Reference		
Female	0.99	0.77	1.27	**1.59**	**0.91**	**2.27**
Race/ethnicity						
Non-Hispanic white	Reference			Reference		
Non-Hispanic black	1.12	0.81	1.54	-0.39	-1.09	0.30
Mexican-American	1.22	0.91	1.64	0.76	-0.08	1.60
Other	1.34	0.85	2.12	0.49	-0.79	1.77
Poverty to income ratio						
< 1	0.80	0.51	1.26	-1.18	-3.36	1.01
≥ 1 < 2	1.10	0.66	1.82	-0.20	-2.29	1.88
≥ 2 < 3	0.86	0.46	1.62	-0.52	-2.70	1.66
≥ 3 < 4	0.53	0.26	1.08	-1.00	-3.34	1.35
≥ 4 < 5	0.66	0.32	1.36	-1.66	-4.01	0.68
≥ 5	Reference			Reference		

*Bolded values indicate significance at P < 0.05.

^† ^n =1340, F statistic = 3.41 (P = 0.009) for the overweight status multivariate Poisson regression model.

^§ ^Skinfold thicknesses = (Subscapular + Triceps skinfolds). n = 1337, r^2 ^= 0.042 (P = 0.0031) for the skinfolds multivariate linear regression model

From the multivariate linear regression model, adjusting for age, gender, race/ethnicity, and income: computer use ( > 0 hours on the assessment day) was associated with higher skinfold thicknesses (Table 6, β = 0.56, 95% CI [0.04, 1.07], P = 0.04). Female gender was also associated with higher skinfold thicknesses (Table 6), while computer use was not associated with weight status (P > 0.05).

**Table 6:** Computer use as a correlate for overweight status and skinfold thicknesses mm).*

	Overweight Status^†^			Skinfolds^§^		
	Prevalence Ratio	[95%	CI]	β	[95%	CI]

Computer use						
0 hrs/day	Reference			Reference		
> 0 hrs/day	1.16	0.89	1.51	**0.56**	**0.04**	**1.07**
Age (years)	1.13	0.99	1.30	0.07	-0.39	0.52
Gender						
Male	Reference			Reference		
Female	0.98	0.76	1.27	**1.58**	**0.87**	**2.29**
Race/ethnicity						
Non-Hispanic white	Reference			Reference		
Non-Hispanic black	1.11	0.81	1.52	-0.43	-1.12	0.26
Mexican-American	1.19	0.88	1.61	0.68	-0.18	1.53
Other	1.32	0.83	2.08	0.31	-0.91	1.53
Poverty to income ratio						
< 1	0.86	0.56	1.32	-0.91	-2.97	1.15
≥ 1 < 2	1.17	0.71	1.91	0.10	-1.89	2.09
≥ 2 < 3	0.87	0.46	1.63	-0.47	-2.61	1.68
≥ 3 < 4	0.53	0.26	1.12	-1.01	-3.39	1.36
≥ 4 < 5	0.69	0.33	1.44	-1.46	-3.68	0.75
≥ 5	Reference			Reference		

*Bolded values indicate significance at P < 0.05. ^† ^n = 1340, F statistic = 1.62 (P = 0.17) for the overweight status multivariate Poisson regression model.

^§ ^Skinfold thicknesses = (Subscapular + Triceps skinfolds). n = 1339, r^2 ^= 0.037 (P = 0.009) for the skinfolds multivariate linear regression model.

From the multivariate linear regression model, adjusting for age, gender, race/ethnicity, and income: media use in excess of 2 hours on the assessment day had a borderline significant association with increased skinfold thicknesses (Table 7, β = 0.85, 95% CI [-0.04, 1.75], P = 0.06). Female gender was also associated with higher skinfold thicknesses (Table 7). Media use for more than two hours on the assessment day was not associated with higher weight status (P > 0.05). We were unable to analyze the above multivariate models stratified by race/ethnicity due to the lack of participants in more than one primary sampling unit for certain covariates.

**Table 7:** Media use as a correlate for overweight status and skinfold thicknesses (mm).*

	Overweight Status^†^			Skinfold Thicknesses^§^		
	Prevalence Ratio	[95%	CI]	β	[95%	CI]

Media use						
≤ 2 hrs/day	Reference			Reference		
> 2 hrs/day	1.21	0.96	1.54	0.85^||^	-0.04	1.75
Age (years)	1.13	0.99	1.30	0.06	-0.39	0.51
Gender						
Male	Reference			Reference		
Female	0.99	0.77	1.27	**1.60**	**0.89**	**2.30**
Race/ethnicity						
Non-Hispanic white	Reference			Reference		
Non-Hispanic black	1.11	0.80	1.54	-0.44	-1.15	0.27
Mexican-American	1.20	0.88	1.62	0.71	-0.15	1.56
Other	1.36	0.86	2.13	0.50	-0.75	1.75
Poverty to income ratio						
< 1	0.83	0.53	1.29	-1.07	-3.20	1.07
≥ 1 < 2	1.11	0.67	1.85	-0.13	-2.18	1.91
≥ 2 < 3	0.86	0.46	1.61	-0.49	-2.64	1.66
≥ 3 < 4	0.53	0.26	1.09	-1.02	-3.39	1.35
≥ 4 < 5	0.68	0.33	1.40	-1.55	-3.84	0.73
≥ 5	Reference			Reference		

*Bolded values indicate significance at P < 0.05.

^† ^n =1337, F statistic = 2.77 (P = 0.025) for the overweight status multivariate Poisson regression model.

^§ ^Skinfold thicknesses = (Subscapular + Triceps skinfolds). n = 1334, r^2 ^= 0.039 (P = 0.008) for the skinfold multivariate linear regression model.

^∥ ^P = 0.061

## Discussion

In a large, population-based survey of children, aged 2–5 years, we report that a substantial proportion of preschoolers exceeded the AAP recommendations to limit media time to less than 2 hours daily. This finding is consistent with previous studies in preschoolers [10,12,13]. Preschoolers had a higher prevalence and greater exposure to television/video viewing than computer use as previously reported [10]. Importantly, we report that TV/video viewing for more than 2 hours per day in this nationally representative sample of US preschoolers was independently associated with being overweight or at risk for overweight and with higher adiposity as measured by skinfold thicknesses. These results update and expand the findings of a previous large study on 3 year old children that used older data from the early to mid 1990s [13] and highlights the importance of television viewing to weight status and adiposity in early childhood.

In contrast, we report that computer use among preschoolers is low, consistent with previous reports [10,19,27,28]. From the bivariate analyses, computer use generally increased with increasing age and income. Additionally, compared to non-Hispanic white children, more non-Hispanic black children were reported to have used a computer on the assessment day while fewer Mexican American children did. These findings build upon a previous survey that reported similar demographic associations for 6 month to 6 year olds with regard to ever having used a computer [28]. It is thought that access to computers at schools [27] coupled with heavier computer use may help explain why previous reports have documented that African-American children used computers either more than [28] or at the same level [19] as white children. Given preschooler's low exposure to computer use, immature motor skills, and the relative lack of age-appropriate software, it was not surprising that preschooler's computer use was low, nor the lack of association with weight status. Surprisingly, any computer use ( > 0 hours per day) was independently associated with higher adiposity, as measured by the sum of triceps and subscapular skinfold thicknesses. The relationship between computer use and adiposity warrants confirmation and further study, especially as the trend for increasing computer use continues among preschool children as more software aimed at preschoolers becomes available. Similar to computer use, the composite measure of media use for more than 2 hours on the assessment day had borderline association with higher adiposity, as measured by the sum of triceps and subscapular skinfold thicknesses (β = 0.85, 95% CI [-0.04, 1.75], P = 0.06), but not with weight status (P > 0.05).

This study has several limitations. First, the cross-sectional nature of this study precludes drawing causal inferences. However, given that the relationship between TV/video viewing and excess weight has been identified by intervention trials in school-age children, it seems plausible that this relationship holds true to some extent in their younger peers. While Dennison and colleagues have previously reported that a preschool-based intervention can reduce TV/video viewing in 2–5 year old children, they were unable to show a difference in change in BMI between the intervention and controls [29]. This lack of change in BMI may be due to the small sample size of this trial–only 77 subjects had complete follow-up data as compared to 192 subjects in Robinson's intervention trial involving 3^rd ^and 4^th ^grade students in which he showed the relationship between television viewing and excess weight gain [5]. Larger, long-term, controlled intervention trials for preschool-age children are necessary to clarify this issue. Second, television viewing and computer use were obtained by single item question and parental report, which may limit their validity [30]. However, previous studies that have compared direct parental estimates of children's television viewing have reported significant correlation with television diaries [31] and showed no systematic bias [32]. Random error would likely bias our findings towards the null hypothesis. Third, the effect sizes of the television/video (r^2 ^= 0.042) or computer (r^2 ^= 0.037) multivariate models were modest, although they were consistent with those from a recent meta-analysis [4], and were not unexpected due to the cross-sectional design of the study. Moreover, since single item, parent recalls were used to assess the television/video and computer exposures, these subjective measures may contribute to the weak associations with adiposity in this study and other studies as previously reviewed [30], rather than there being a true small effect size. Fourth other forms of media use such as video game console playing were not assessed. Moreover, combining television/video viewing with computer use likely underestimated true media use since we took a conservative approach and classified those participants who reported less than 1 hour of television or computer use as having none. This approach may also bias our findings for overall media use towards the null hypothesis, and help explain why we found only borderline association between media use and adiposity and no association with weight status. Finally, the survey provides no information on the content of media use, and so we cannot ascertain which types of programs or advertisements are associated with higher weight status and adiposity.

## Conclusion

This study confirms that a substantial percent (almost 36%) of US preschool children exceeded the AAP recommendation to limit media time to 2 hours or less per day. The majority of media time was spent on television/video viewing rather than computer use. Moreover, almost 31% of preschool children exceeded the AAP recommendation by television/video viewing alone. This study provides support using recent, nationally representative data for the AAP's recommendation to limit television/video viewing with regard to obesity and adiposity in US preschoolers. Intervention studies to prevent and treat obesity in preschool children by reducing TV/video viewing are warranted. Further research is necessary to determine what mediates the relationship between TV/video viewing and a child's weight status. This study is the first to report a relationship between computer use among preschool children and higher adiposity. More studies are necessary to confirm and explore this relationship.

## Abbreviations

AAP = American Academy of Pediatrics, NHANES = National Health and Nutrition Examination Survey, CDC = Centers for Disease Control and Prevention, BMI = body mass index, PIR = poverty to income ratio, SE = standard error, CI = Confidence Interval

## Competing interests

The author(s) declare that they have no competing interests.

## Authors' contributions

JAM participated in the study's conception and design; led the analysis and interpretation of data; and drafted the manuscript. FJZ participated in the design, analysis, and interpretation of data and helped to draft the manuscript. DAC participated in the conception, design, analysis, and interpretation of data and helped to draft the manuscript. All authors read and approved the final manuscript.
